# The possibility of prognostic and functional values of the 8q24 and 20q13 chromosomal bands in colorectal cancer

**DOI:** 10.22099/mbrc.2025.54114.2202

**Published:** 2026

**Authors:** Seyed Ahmadreza Siadat

**Affiliations:** Department of Biology, School of Science, Shiraz University, Shiraz, Iran

**Keywords:** Chromosomal Bands, Colorectal Cancer, Enrichment Analysis, Genome-Wide Association Studies, Polymorphism

## Abstract

Colorectal cancer (CRC) remains a major global health concern, especially given its increasing incidence among younger individuals. While genome-wide association studies (GWAS) have identified numerous CRC-associated polymorphisms, their spatial distribution and functional implications are not fully understood. This study examined the locations of 1,346 CRC-linked polymorphisms across chromosomal bands. The results revealed significant nonrandom clustering across thirteen chromosomal bands: 1q41, 6p21, 8q24, 9q34, 10p14, 10q25, 11q12, 12p13, 15q13, 18q21, 19q13, 20p12, and 20q13. Functional enrichment analysis of genes within these bands revealed several Gene Ontology (GO) terms and Kyoto Encyclopedia of Genes and Genomes (KEGG) pathways. Reciprocal chromosomal enrichment confirmed that many of these terms and pathways were not randomly localized within the same bands, highlighting their potential biological significance. Survival analysis using TCGA data identified three KEGG pathways and 33 GO terms mapped to nine of the thirteen bands that were significantly associated with poor prognosis. Notably, the 8q24 and 20q13 regions were enriched for differentially expressed genes and survival-associated terms yet showed no significant enrichment for genes with high somatic mutation rates. These results imply that 8q24 and 20q13 act as regulatory hotspots rather than mutation-driven regions. Overall, this integrative approach identified functionally and clinically relevant genomic regions that may contribute to inherited CRC risk and progression, providing valuable targets for the development of diagnostic and prognostic biomarkers.

## INTRODUCTION

Colorectal cancer (CRC) is a common malignancy and the second leading cause of cancer-related deaths worldwide. Despite advances in prevention, screening, and treatment, the incidence of CRC is increasing, particularly among younger individuals. This highlights the need for a better understanding of its genetic landscape [1]. Genome-wide association studies (GWAS) have identified numerous genetic variants associated with CRC risk, shedding light on inherited predispositions [2-4]. However, although these individual risk loci have been extensively cataloged, the functional significance, spatial distribution, and collective impact of these variants across chromosomal regions remain poorly understood. This knowledge gap hinders their translation into clinical applications. Investigating the genomic organization and chromosomal localization of these polymorphisms could reveal nonrandom clustering patterns and highlight regulatory hotspots involved in tumorigenesis.

Previous studies have demonstrated that the nonrandom distribution of cancer-associated polymorphisms may indicate chromosomal regions containing key oncogenes, tumor suppressors, or elements involved in gene regulation [5-7]. Other studies have shown that alterations in specific chromosomal bands can affect gene expression, disrupt cellular pathways, and promote malignant transformation [8, 9]. Despite these observations, a comprehensive analysis of chromosomal bands enriched for CRC-associated polymorphisms and their functional implications is lacking. Therefore, the present study sought to address this gap.

## MATERIALS AND METHODS

**Figure 1:**
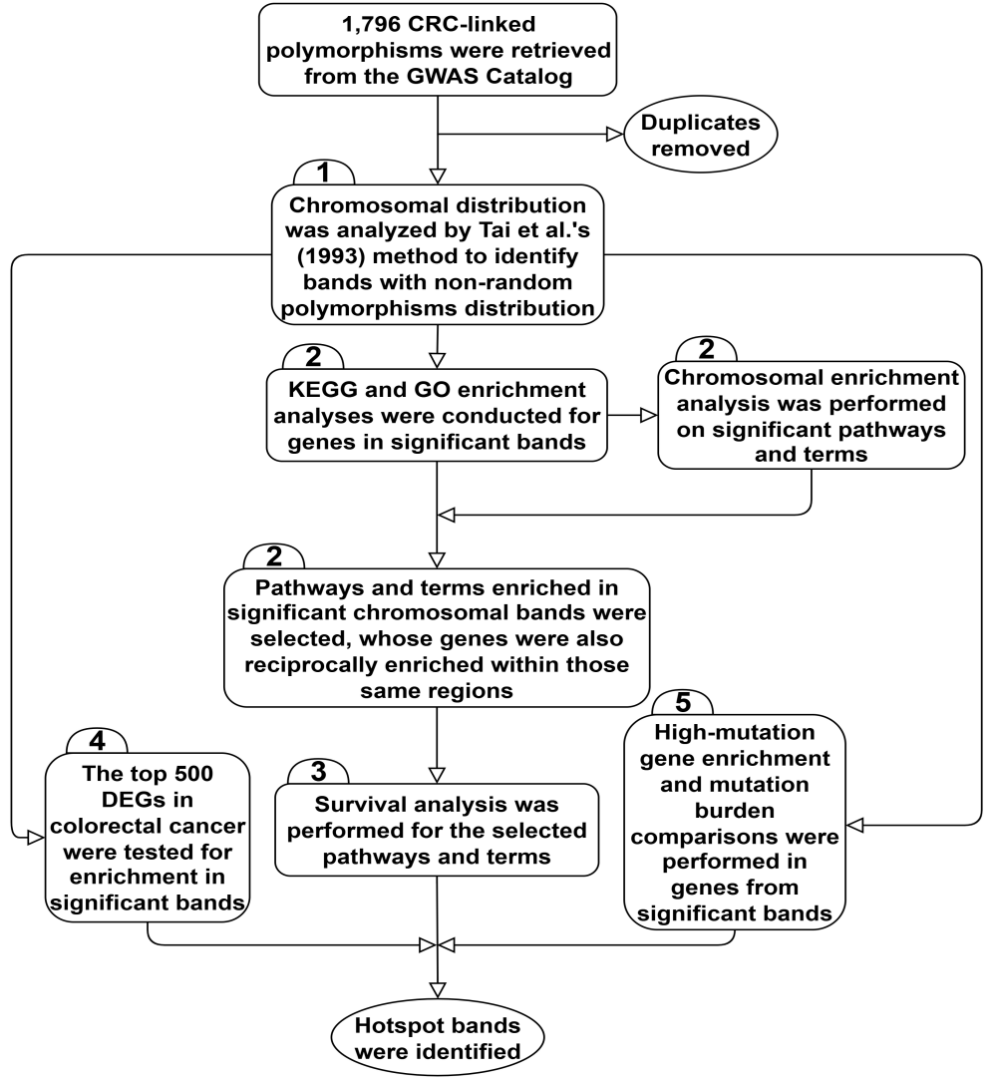
Diagram illustrating the overall workflow employed in the study.

The current study consisted of five steps (Fig. 1). They are described below.

### Step 1: Chromosomal Distribution of CRC-Associated Polymorphisms:

On July 12, 2024, we obtained the polymorphisms from the MONDO_0005575 dataset, consisting of 75 GWAS on colorectal cancer (see Supplementary Data). We subsequently evaluated the nonrandom distribution of these polymorphic sites across human chromosomes [10].

### Step 2: Enrichment Analyses:

Two enrichment analyses were performed in this step. First, we used the biomaRt package in R to extract all genes located within chromosomal bands harboring nonrandom CRC-associated polymorphic sites from Ensembl. We then performed Gene Ontology (GO) and Kyoto Encyclopedia of Genes and Genomes (KEGG) pathway enrichment analyses on these gene sets using the clusterProfiler package in R. The GO terms were categorized into biological processes (BP), molecular functions (MF), and cellular components (CC). Second, we used the AnnotationDbi and KEGGREST packages to retrieve genes associated with each GO term and KEGG pathway, respectively. Finally, we conducted a chromosomal location enrichment analysis of these genes using the msigdbr package.

### Step 3: Survival Analysis:

Survival analysis was conducted using the cSurvival web tool (https://tau.cmmt.ubc.ca/cSurvival) [11]. based on the TCGA-COAD and TCGA-READ datasets. Kaplan-Meier estimates and log-rank tests were used to analyze the relationship between KEGG pathways and GO terms expression levels and overall survival.

### Step 4: Differentially Expressed Genes (DEGs) Analysis:

The top 500 differentially expressed genes from TCGA-COAD, including 250 upregulated and 250 downregulated genes, were obtained via UALCAN (https://ualcan.path.uab.edu/index.html) [12]. Enrichment of these DEGs within chromosomal bands was assessed using hypergeometric testing.

### Step 5: Mutation Burden Analysis in Chromosomal Bands:

Somatic mutation data for COAD and READ were obtained from The Cancer Genome Atlas (TCGA) via the TCGAbiolinks package. Mutations within coding sequences (CDS) were normalized using the following formula: 

$\mathrm{Adjusted Mutation Count}=\frac{\mathrm{Total Mutations}}{\mathrm{CDS Length}+\mathrm{Q1}}\times \log_{2}\left(\mathrm{CDS Length}\right)$

where Q1 represents the first quartile of gene lengths, mitigating gene size bias. Gene length information was obtained from Ensembl using the biomaRt package. This adjustment accounts for gene size while emphasizing the relative mutation burden in longer genes. Genes with adjusted mutation counts above the median were classified as high-mutation genes. A hypergeometric test was used to assess their enrichment within chromosomal bands. Moreover, the mean adjusted mutation burden of genes in each band was compared with background genes using a *t*-test.

### Statistical Analyses and Data Visualization:

Statistical analyses were conducted in R, and significant bands, along with their polymorphism burden, were visualized using the chromPlot package. To account for multiple hypothesis testing and control the false discovery rate, the Benjamini-Hochberg method was used to adjust p-values. Statistical significance was defined as an adjusted *p*-value less than 0.05.

## RESULTS

A total of 1,796 CRC-associated polymorphisms were compiled from 75 GWAS studies. After removing duplicates, 1,346 unique variants were retained for further analysis. Of these variants, 637 were located within protein-coding genes, with the majority (577) occurring in intronic regions. The remaining 709 variants were mapped to non-coding regions, including 503 in unannotated intergenic elements. To investigate their genomic distribution, we examined the location of these variants across chromosomal bands. The results revealed significant nonrandom clustering across thirteen chromosomal bands (1q41, 6p21, 8q24, 9q34, 10p14, 10q25, 11q12, 12p13, 15q13, 18q21, 19q13, 20p12, and 20q13) (Table 1, Figure S1).

**Table 1:** Chromosomal distribution of colorectal cancer-associated polymorphisms

**Chromosomal band**	**Number of polymorphic sites**	**F**	**df**	**Adj p-Value**
1q41	18	4.55	2658, 36	5.59 × 10⁻⁵
6p21	27	4.15	2640, 54	4.66 × 10⁻⁷
8q24	32	2.71	2630, 64	2.46 × 10⁻⁴
9q34	18	3.96	2658, 36	3.83 × 10⁻⁴
10p14	10	4.27	2674, 20	4.18 × 10⁻²
10q25	18	3.25	2658, 36	5.46 × 10⁻³
11q12	13	3.99	2668, 26	9.49 × 10⁻³
12p13	21	3.46	2652, 42	4.70 × 10⁻⁴
15q13	18	7.73	2658, 36	1.89 × 10⁻⁸
18q21	20	2.66	2654, 40	2.90 × 10⁻²
19q13	26	2.34	2642, 52	2.59 × 10⁻²
20p12	27	5.09	2640, 54	5.92 × 10⁻⁹
20q13	29	3.28	2636, 58	1.88 × 10⁻⁵

**Note:** total of 1,346 CRC-associated polymorphisms were analyzed and bands that bearing non-randomly polymorphic sites were shown in the Table.

Next, we performed two enrichment analyses. First, we retrieved all genes located within the aforementioned bands and subjected them to enrichment analysis to elucidate their functional significance. This analysis revealed a total of 207 GO terms and 10 KEGG pathways, which are summarized in Tables S1 and S2, respectively. We know that genes involved in biological pathways are located on different chromosomes. Second, to investigate whether the genes associated with the aforementioned KEGG pathways and GO terms are nonrandomly distributed and are primarily located within the thirteen specified chromosomal bands, we conducted an additional analysis. We retrieved all genes associated with these pathways and terms and tested them for enrichment within the previously identified chromosomal bands (see Tables S3 and S4). This analysis revealed that all chromosomal bands, except 10p14, were significantly enriched for genes associated with at least one respective KEGG pathway and/or GO term. These results suggest the functional relevance of these chromosomal bands.

Following the previous enrichment analyses, in the third step we performed survival analysis on the KEGG pathways and GO terms that were mutually enriched within the aforementioned 12 chromosomal bands. This analysis evaluated the clinical relevance of our findings. The goal was to determine whether functionally enriched GO terms and KEGG pathways were associated with patient prognosis. The analysis identified three KEGG pathways and 33 GO terms that were significantly associated with survival in CRC (Table 2). Notably, these prognostic pathways and terms were associated with nine chromosomal bands: 8q24, 9q34, 10q25, 11q12, 12p13, 15q13, 18q21, 19q13, and 20q13.

In the fourth step of the present study, we examined the randomness distribution of the top 500 DEGs from the COAD project on the chromosomal bands associated with CRC that we identified in the first step. Our analysis revealed that only two chromosomal bands, 8q24 and 20q13, were significantly enriched with the DEGs (Table 3). 

Lastly, we examined the somatic mutation burden of genes located on chromosomal bands associated with CRC. These bands were identified in the first step of the study. However, no statistically significant association was found between somatic mutation burden and chromosomal bands (Table S6).

## DISCUSSION

The present study identified thirteen chromosomal bands that exhibiting a non-random distribution of CRC-associated GWAS polymorphisms. This finding suggests the functional relevance of these chromosomal bands in CRC. These regions have previously been associated with cancer progression. For example, 8q24 contains regulatory elements that control MYC expression [13, 14].

**Table 2:** Survival analysis of KEGG pathways and Gene Ontology terms bidirectionally enriched in chromosomal bands

**Category**	**Term ID**	**Description**	**Chromosomal band**	**Adj p-value**
MF	GO:0001786	Phosphatidylserine binding	8q24	4.46 × 10⁻²
MF	GO:0033130	Acetylcholine receptor binding	8q24	1.09 × 10⁻²
CC	GO:0005930	Axoneme	9q34	4.46 × 10⁻²
CC	GO:0036126	Sperm flagellum	9q34	2.45 × 10⁻²
BP	GO:0070085	Glycosylation	9q34	4.46 × 10⁻²
CC	GO:0097014	Ciliary plasm	9q34	1.09 × 10⁻²
MF	GO:0004935	Adrenergic receptor activity	10q25	2.45 × 10⁻²
KEGG	hsa04740	Olfactory transduction	11q12	1.63 × 10⁻²
BP	GO:0001580	Detection of chemical stimulus involved in sensory perception of bitter taste	12p13	4.46 × 10⁻²
BP	GO:0001906	Cell killing	12p13	2.70 × 10⁻²
MF	GO:0008527	Taste receptor activity	12p13	6.55 × 10⁻³
MF	GO:0033038	Bitter taste receptor activity	12p13	2.45 × 10⁻²
BP	GO:0050909	Sensory perception of taste	12p13	1.09 × 10⁻²
BP	GO:0050912	Detection of chemical stimulus involved in sensory perception of taste	12p13	4.46 × 10⁻²
BP	GO:0050913	Sensory perception of bitter taste	12p13	4.46 × 10⁻²
CC	GO:0070820	Tertiary granule	12p13	2.41 × 10⁻²
CC	GO:0070821	Tertiary granule membrane	12p13	4.46 × 10⁻²
MF	GO:0140375	Immune receptor activity	12p13	4.46 × 10⁻²
KEGG	hsa04742	Taste transduction	12p13	1.63 × 10⁻²
CC	GO:0000137	Golgi cis cisterna	15q13	1.63 × 10⁻²
MF	GO:0005231	Excitatory extracellular ligand-gated monoatomic ion channel activity	15q13	1.09 × 10⁻²
MF	GO:1904315	Transmitter-gated monoatomic ion channel activity involved in regulation of postsynaptic membrane potential	15q13	4.46 × 10⁻²
KEGG	hsa05033	Nicotine addiction	15q13	3.76 × 10⁻²
MF	GO:0004857	Enzyme inhibitor activity	18q21	4.46 × 10⁻²
BP	GO:0043086	Negative regulation of catalytic activity	18q21	4.46 × 10⁻²
MF	GO:0001228	DNA-binding transcription activator activity, RNA polymerase II-specific	19q13	1.72 × 10⁻²
BP	GO:0002765	Immune response-inhibiting signal transduction	19q13	6.55 × 10⁻³
MF	GO:0004875	Complement receptor activity	19q13	4.46 × 10⁻²
MF	GO:0032396	Inhibitory MHC class I receptor activity	19q13	4.77 × 10⁻²
MF	GO:0033691	Sialic acid binding	19q13	4.46 × 10⁻²
CC	GO:0070821	Tertiary granule membrane	19q13	4.46 × 10⁻²
MF	GO:0140375	Immune receptor activity	19q13	4.46 × 10⁻²
MF	GO:0004857	Enzyme inhibitor activity	20q13	4.46 × 10⁻²
BP	GO:0006959	Humoral immune response	20q13	4.46 × 10⁻²
BP	GO:0019730	Antimicrobial humoral response	20q13	3.51 × 10⁻²
BP	GO:0043086	Negative regulation of catalytic activity	20q13	4.46 × 10⁻²

**Note:** The full table is provided in Table S5.

**Table 3:** Chromosomal enrichment analysis of differentially expressed genes (DEGs) in CRC-associated chromosomal bands

**Chromosomal bands**	**Number of genes in each band**	**Number of DEGs in each band**	**Adj p-value**
**1q41**	36	2	1.00
**6p21**	277	7	1.00
**8q24**	170	11	8.50 × 10⁻³
**9q34**	211	4	1.00
**10p14**	16	0	1.00
**10q25**	56	1	1.00
**11q12**	204	4	1.00
**12p13**	187	3	1.00
**15q13**	33	0	1.00
**18q21**	81	3	1.00
**19q13**	780	15	1.00
**20p12**	41	0	1.00
**20q13**	217	14	1.69 × 10⁻³

**Note:** The comparison was performed assuming a total gene count of 25,000 and a total of 500 DEGs. Significant enrichment of DEGs within chromosomal bands is indicated by adjusted p-values less than 0.05.

These regions may play critical roles in CRC pathogenesis by harboring key regulatory elements, tumor suppressor genes, or oncogenes. As previously suggested, genetic polymorphisms in certain chromosomal regions could be leveraged to develop a laboratory diagnostic test for mass screening programs, enabling the identification of individuals at high risk for various diseases [15, 16]. Similarly, genetic variations in the 13 chromosomal regions identified in this study could facilitate the development of diagnostic kits for colorectal cancer.

Survival analysis revealed that three KEGG pathways and 33 GO terms, associated with nine chromosomal bands, were significantly linked to poor CRC survival (Table 2). Many of these pathways and terms, such as those involved in olfactory and taste receptor signaling, immune regulation, microRNA (miRNA) transcription control, and enzyme inhibition, have been previously linked to CRC processes, including tumor growth, immune evasion, and microenvironmental remodeling [17-23]. These results suggest that these chromosomal bands and their associated pathways could serve as indicators of CRC prognosis.

The present study showed that the 8q24 and 20q13 chromosomal bands were enriched for DEGs and six GO terms associated with CRC patient survival. The 8q24 region, which harbors the MYC oncogene, plays a central role in cell proliferation, and its amplification has been linked to increased CRC susceptibility [13, 24]. Similarly, 20q13 is involved in the disruption of cell cycle regulation and apoptosis, with recurrent amplifications and deletions contributing to tumorigenesis [25-27]. Notably, amplification of the 20q13.33 sub-band has been reported as an early and frequent event in sporadic colorectal cancer and proposed as a potential biomarker for early tumor detection [26]. Importantly, neither of these regions showed enrichment for genes with high somatic mutation rates, and their average mutation burden was comparable to background levels. These findings support the idea that 8q24 and 20q13 primarily act as transcriptional regulatory hotspots rather than being involved in functional alterations due to somatic mutations.

Collectively, this study revealed a nonrandom distribution of CRC-associated GWAS polymorphisms across thirteen chromosomal bands. Enrichment analyses highlighted 8q24 and 20q13 as regulatory hotspots, enriched for differentially expressed genes and survival-associated pathways, but not for highly mutated genes. These findings underscore the functional and prognostic importance of these regions and suggest their potential utility as genetic biomarkers for CRC diagnosis, prognosis, and population-level screening.

### Acknowledgements:

 I wish to express my sincere gratitude to Professor Mostafa Saadat for his invaluable guidance and support, which were instrumental in the advancement of this research.

### Conflict of Interest:

 The author declares no conflicts of interest.

### Ethics approval and consent to participate:

Not required. 

### Authors’ Contribution:

SARS: Conceptualization, Methodology, Investigation, Formal Analysis, Data Curation, Visualization, Validation, and Writing.
